# Impact of the COVID-19 Pandemic on Trends in Cardiothoracic Imaging

**DOI:** 10.1155/2022/7923228

**Published:** 2022-06-16

**Authors:** Kathleen M. Capaccione, Sophia Huang, Jay S. Leb, Belinda D'souza, Jonathan Goldstein, Mary M. Salvatore

**Affiliations:** Department of Radiology, Columbia University Irving Medical Center, 622 W 168 Street, New York, NY 10032, USA

## Abstract

**Introduction:**

Here, we evaluate the effect of the COVID-19 pandemic on utilization of cardiothoracic imaging studies.

**Methods:**

We queried our radiology record system to retrospectively identify numbers of specific key cardiothoracic imaging studies for five years prior and during the COVID-19 pandemic. Statistical analysis was performed to evaluate changes in the number of exams in 2020 and 2021 compared to 2019.

**Results:**

Five-year retrospective analysis demonstrated progressive increases in nearly all cross-sectional studies. In 2020, daily chest radiograph utilization decreased with an overall number of daily radiographs of 406 (SD = 73.1) compared to 480 per day in 2019 (SD = 82.6) (*p* < 0.0001). Portable radiograph utilization was increased in 2020 averaging 320 (SD = 68.2) films daily in 2020 compared to 266 (SD = 29.1) in 2019 (*p* < 0.0001). Utilization of thoracic CT was decreased during the pandemic, with 21.8 (SD = 12.9) studies daily compared to 52.0 (SD = 21.4) (*p* < 0.0001) studies daily in 2019. Cardiac imaging utilization was also substantially decreased in 2020 compared to 2019, averaging a total of 3.8 (SD = 3.2) versus 10.8 (SD = 6.6) studies daily and 0.88 (SD = 1.7) versus 2.5 (SD = 2.3) studies daily for CT and MRI, respectively. Evaluation of cardiothoracic imaging for the subsequent 18 months after New York's entry to phase I recovery in June 2020 demonstrated that by one year after the emergence of COVID-19 imaging utilization had recovered to prepandemic levels. Cardiac imaging continued to increase throughout the chronic phase of the COVID-19 pandemic, reaching almost twice the prepandemic levels by the end of 2021.

**Conclusion:**

COVID-19 has had far-reaching effects on medicine and public health. Here, we demonstrate decreases in all cross-sectional cardiothoracic imaging studies, closely mirroring findings in other fields during the height of the pandemic, which have since rebounded.

## 1. Introduction

Cardiothoracic imaging encompasses the most commonly ordered radiology studies and is a cornerstone of medical decision-making [1]. Cardiothoracic imaging utilization has increased over the past decade [2], and research has focused on the increasing use of computed tomography angiography for pulmonary embolism (CTA-PE) in the emergency setting [3]. CTA for aortic dissection [4] and coronary artery disease [5] have seen parallel increases over time [6]. Cardiac MRI is less commonly obtained [7], but utilization has similarly continued to increase with increasing availability and a growing number of indications [8]. Without imposition of regulatory limits or external pressures limiting imaging utilization, advances in imaging quality and techniques promote the continued increase of cardiothoracic imaging utilization.

The COVID-19 pandemic has been an unprecedented event in modern healthcare, infecting millions worldwide [9]. Chest computed tomography (CT) is sensitive but not specific for the diagnosis of COVID-19 infection [10, 11]. The Society for Thoracic Radiology (STR) issued a position statement advising against the routine use of chest CT for the diagnosis of COVID-19 but recommended its use to assess complicating conditions such as pulmonary abscess [12]. The American College of Radiology (ACR) recommended against the routine use of chest CT in patients suspected of COVID-19 reflecting the ACR appropriateness criteria for acute respiratory illness which states that chest CT is “usually not appropriate” [13].

The overall effect of the COVID-19 pandemic on trends in cardiothoracic imaging has, to this point, been unclear; cardiothoracic radiologists practicing during the pandemic reported a significant decline in cardiac imaging, likely resulting from the prohibition on elective procedures and patient preference to remain home and avoid exposure [14]. Conversely, those working in the height of the pandemic reported a significant increase in chest radiograph utilization. Thrombotic complications of COVID-19 led to frequent clinical concern for pulmonary embolism in patients with COVID-19 and patients often underwent CTA-PE [15].

Here, we sought to quantify imaging utilization for key cardiothoracic imaging studies during the pandemic and evaluate how they are related to trends over time in order to better understand the effect of the COVID-19 pandemic on trends in cardiothoracic imaging utilization.

## 2. Materials and Methods

We obtained a Human Research Protection Office and Internal Review Board Letter of Exemption to retrospectively study imaging trends over time; exemption was granted given that no individual patient data were accessed. We queried the radiology record system “MModal Catalyst” for all reports with specific exam labels corresponding with the type of study being evaluated regardless of age or gender. Imaging studies, rather than billing data/ICD-10 data, were used given that diagnoses were not always known at the time of admission, and therefore these data were the most accurate representation of the studies obtained. Data were recorded as yearly totals from 2015 to 2019 and daily totals for the period of the pandemic spanning from 03/11/2020 to 06/08/2020. Subsequently, monthly data were obtained from 06/01/2020 to 12/31/2021 and normalized to average monthly values prior to the pandemic, collected from 06/01/2019 to 12/31/2019. Sample size for this study was not prespecified given that all exams in the time frame were included in the analysis.

All statistical analyses were performed in Microsoft Excel and using online calculator “Statistics for the Social Sciences” [16]. Statistical analysis was performed in conjunction with the Department of Biostatistics at the Mailman School of Public Health. Data were tested to assess if they followed a normal distribution; some datasets did, while others did not. Averages and standard deviations (SDs) were calculated for each sample, as well as the median value. For samples following a normal distribution, two-tailed paired Student's *t*-test was used to assess statistical significance between samples. For samples that did not follow a normal distribution, we tested for significant differences using a Wilcoxon signed-rank test. For yearly numbers, data were normalized to 2015 exam numbers and expressed as a percent change. Of note, for all types of examinations, there was substantial variation in the quantity of imaging studies obtained on weekend days compared to weekdays which contributed significantly to standard deviation.

## 3. Results

### 3.1. Five-Year Trends in Cardiothoracic Imaging Utilization

**Figure 1:**
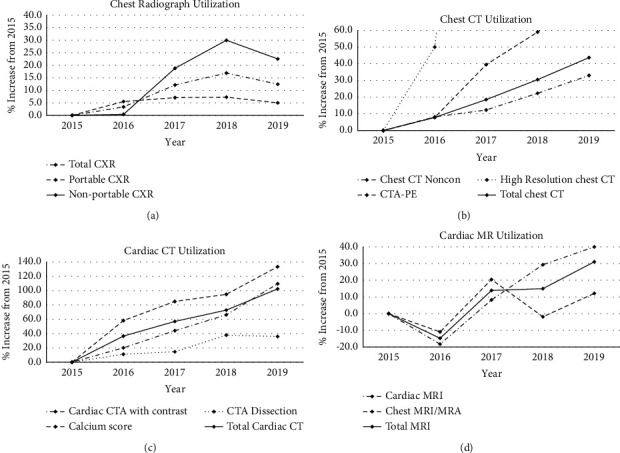
Five-year trends in utilization of key cardiothoracic imaging exams: (a) chest radiograph, (b) chest CT, (c) cardiac CT, and (d) cardiac MRI. In all cross-sectional modalities, there was a progressive increase in the number of studies over time. Chest radiograph utilization progressively increased over time although it demonstrated a small decline in 2019.

We first sought to establish trends in cardiothoracic imaging within our practice, comprised a 900-bed university hospital in northern Manhattan and two affiliate community hospitals. Given the wide variety in average numbers of each type of exam, trends are expressed as percent change compared to 2015.  Figure 1(a) demonstrates the five-year trend in overall, portable, and nonportable chest radiographs.  Figure 1(b) demonstrates trends in thoracic CT studies, including noncontrast chest CT, CTA-PE, and high-resolution chest CT (HRCT).  Figure 1(c) shows trends in cardiac CT studies, including cardiac CTA, CT for calcium scoring, CTA for transcatheter aortic valve replacement (TAVR), and CTA for dissection.  Figure 1(d) demonstrates trends in cardiac MRI. Demographic information was not collected given that only numbers of exams were analyzed.

### 3.2. Impact of the COVID-19 Pandemic on Cardiothoracic Imaging Utilization

**Figure 2:**
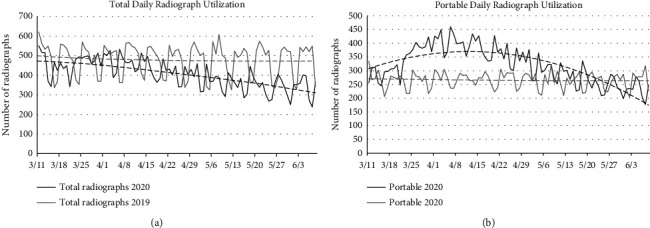
Chest radiograph utilization. (a) Daily chest radiograph utilization for 2020 and 2019, respectively. In 2020, there were statistically significantly fewer radiographs obtained, and there was a trend of decreased utilization throughout the course of the pandemic. (b) Daily trends in portable chest radiograph utilization for 2020 and 2019, showing increasing use as the pandemic peaked and then declining as fewer patients were hospitalized. Dashed lines represent polynomial lines of best fit.

The WHO officially declared the SARS-CoV-2 coronavirus pandemic on March 11, 2020. This also marked the beginning of the height of COVID-19 cases in the greater New York City region, which continued until June 8, when New York entered phase I of the reopening plan. We evaluated daily chest radiograph utilization during this time by analyzing numbers of chest radiographs (Figure 2(a)). Statistically significantly fewer radiographs were performed per day in 2020 during the time frame specified above compared to 2019, averaging 406 (SD = 73.1) and 480 per day (SD = 82.6), respectively (*p* < 0.0001). The average number of portable radiographs was 320 (SD = 68.2) in 2020 compared to 266 (SD = 29.1) in 2019 which also showed a statistically significant difference (*p* < 0.0001) (Figure 2(b)); Table 1 summarizes these values as well as values for other study types discussed below.

**Figure 3:**
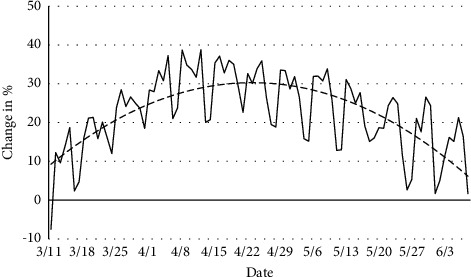
Daily change in % portable chest radiograph in 2020 compared to 2019. Polynomial line of best fit indicates that the peak of portable radiograph utilization coincided with the height of the pandemic in our region. Dashed line represents polynomial line of best fit.

In 2020, an average of 78.8% of chest radiographs were obtained using the portable technique; in 2019, 55.4% were portable, again representing a statistically significant difference (*p* < 0.0001). By calculating the difference in percent portable radiograph utilization in 2020 compared to 2019 (Figure 3), we found that every day except March 11 (−7%), there was an increase in the percent portable radiographs obtained, ranging from 1.7 to 40.0%, and this difference peaked at the height of the pandemic when a large number of patients were hospitalized.

**Figure 4:**
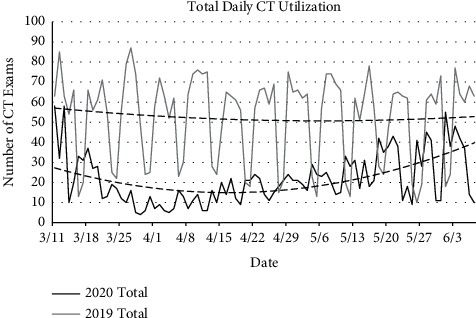
Total daily thoracic CT utilization. At the height of the pandemic, there was greater than 100% decrease in overall daily chest CT utilization. The decline in utilization was inversely related to the number of hospitalized COVID-19 cases, and it increased as fewer patients were hospitalized. Dashed lines represent polynomial lines of best fit.

Similar analysis was performed to evaluate CT utilization. Assessing total thoracic CTs, in 2020, the daily average number of CTs obtained was 21.8 (SD = 12.9) compared to 2019 with an average daily number of 52.0 thoracic CTs (SD = 21.4), a statistically significant difference (*p* < 0.0001) (Figure 4). In 2019, an average of 37.7 (SD = 18.9) noncontrast chest CTs was obtained daily, compared to 2020 which averaged 14.3 (SD = 11.1) daily; this difference was statistically significant (*p* < 0.0001). Analysis of contrast-enhanced CTA-PE demonstrated an average of 14.1 (SD = 4.4) studies obtained daily in 2019 compared to 7.5 (SD = 4.4) in 2020, also statistically significantly different (*p* < 0.0001). No high-resolution chest CTs were performed in 2020 during the dates queried; 17 were performed during this time period in 2019.

**Figure 5:**
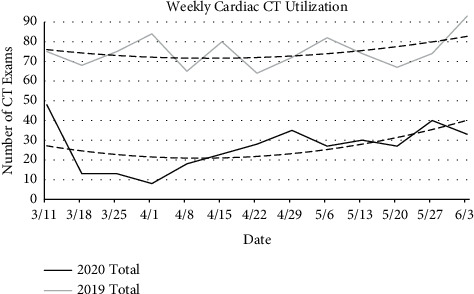
Weekly cardiac CT utilization in 2020 and 2019. Analysis showed a significantly decreased number of cardiac CT studies in 2020 during the COVID-19 pandemic, with the greatest decrease early in the study period. Dashed lines represent polynomial lines of best fit.

Cardiac CT studies were far fewer in number, however showed similar diminution in use in 2020 compared to 2019. The average daily number of all cardiac CT studies included in this analysis (cardiac CT, CT for calcium score, and CTA for dissection) in 2019 was 10.8 (SD = 6.6) compared to an average of 3.8 (SD = 3.2) in 2020, a statistically significant difference (*p* < 0.0001). Given the large daily variation, we analyzed weekly trends in the number of studies obtained in 2020 compared to 2019 (Figure 5). The biggest difference in daily studies obtained was in studies for calcium scoring, with an average of 5.6 (SD = 4.5) daily in 2019 compared to 0.5 daily (SD = 1.0) in 2020 (*p* < 0.0001). A statistically significant decrease in the number of CTA studies for aortic dissection was also seen in 2020 with a total number of 8 studies obtained over the timeframe, compared to 142 in 2019 (daily averages of 0.08 and 1.6 for 2020 and 2019, respectively; *p* < 0.0001). Interestingly, the number of cardiac CTA studies obtained was similar in 2020 compared to 2019, with daily average exam numbers of 3.2 (SD = 2.5) and 3.7 (SD = 3.1), respectively (*p* = 0.22).

**Figure 6:**
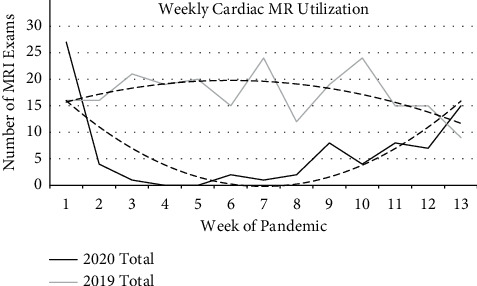
Weekly cardiothoracic MR utilization. Overall cardiothoracic MR imaging utilization during the study period, with a dramatically decreased number of exams obtained in 2020 compared to 2019. Dashed lines represent polynomial lines of best fit.

Cardiac MRI studies demonstrated even more dramatic decreases in utilization in 2020 compared to 2019. Total daily number of cardiac MRI exams averaged 2.5 (SD = 2.3) in 2019, compared to 0.88 (SD = 1.7) in 2020; while there was substantial variation in daily numbers, the difference was nonetheless statistically significant (*p* < 0.0001). Similar to cardiac CT, weekly numbers were analyzed to normalize large daily variation (Figure 6). Numbers of both cardiac and chest MRI studies were decreased in 2020 compared to 2019. In 2020, 64 cardiac MRI exams were obtained compared to 163 during the same time period in 2019, and 15 chest MRI exams were obtained compared to 62. Similar to cardiac CT, MRI utilization was best visualized in terms of weekly trends given large daily variation (Figure 6).

**Figure 7:**
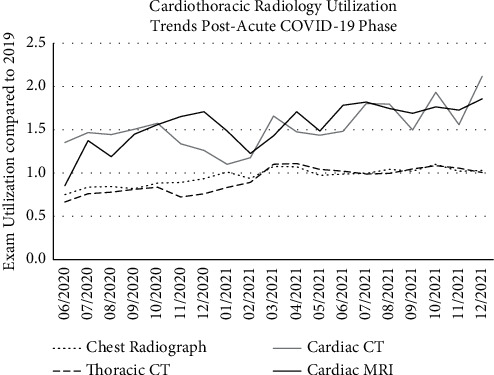
Cardiothoracic imaging utilization after the acute phase of the COVID-19 pandemic. Our data demonstrate that, by early 2021, utilization of all types of cardiothoracic exams had returned to prepandemic levels. Furthermore, both cardiac CT and cardiac MRI utilization exceeded prepandemic levels after recovering from the acute phase of the COVID-19 pandemic and have continued to increase despite the ongoing global health crisis. By the end of 2021, use of some exams was close to 200% of prepandemic levels.

Given that the COVID-19 pandemic persisted beyond phase I recovery, we sought to characterize how cardiothoracic imaging recovered and to evaluate the continued effects of the COVID-19 pandemic on imaging trends. We considered four groups: total chest radiographs, total chest CTs, total cardiac CTs, and total cardiac MRI studies for analysis. Our analysis demonstrated that there was no lasting diminution of cardiothoracic imaging utilization for any of these groups. Chest radiograph and chest CT utilization took about 6 months after entry into phase I recovery to return to their prepandemic levels and then remained there until present. Cardiac CT and cardiac MRI utilization began to increase above prepandemic levels prior to entry into phase I of recovery in the New York region and continued this upward trend, ending 2021 with approximately twice as many exams as performed on average per month prior to the pandemic (Figure 7).

## 4. Discussion

Our data, consistent with other groups, demonstrate progressively increasing utilization of cardiothoracic imaging studies over time. Specific to our practice, exams have steadily increased over time due to an increased patient census as well as expansion of specialty programs such as an interstitial lung disease program and a robust lung cancer practice. Slightly decreased numbers in 2019 prior to the pandemic may have represented leveling off of new patients in these programs.

The COVID-19 pandemic dramatically impacted healthcare, including cardiothoracic imaging utilization. Here, we present the first report to our knowledge that quantitates trends in cardiothoracic imaging during this time. While historic data from the prior five years demonstrated continued increases in the number of cross-sectional studies obtained at our institution, the onset of the pandemic coincided with dramatic decreases in cardiothoracic studies performed. Our data demonstrate decreased chest radiograph utilization in total and significantly increased portable studies. These data concur with recommendations against the regular use of radiography for assessment of COVID-19 [12, 13], but routine use of radiography in the critical care setting, which is almost exclusively portable radiography [17]. Regional data on total hospitalizations indicate peak admissions during the first week of April [18], coinciding with the peak of portable radiography usage. A systematic analysis demonstrated that approximately 30% of admitted COVID-19 patients required ICU care [19], which would account for the high portable radiography utilization during that time. During the pandemic, multiple ICUs were created at our institution to handle the large number of intubated and critically ill patients which expanded the hospital's overall and critical care capacity, contributing to the observed increase in portable radiography.

In contrast, we found that numbers of cross-sectional modalities were significantly decreased in 2020 compared to 2019. For cardiac CT and MRI, these data corresponded to reports of decreased presentations for cardiac symptoms and decline in the use of cardiac catheterization [14]. While the use of cardiac MRI has been suggested for myocarditis secondary to COVID-19 infection [20] and research has characterized associated imaging patterns [21], this indication has reached only limited clinical utility. A study of non-COVID-19 hospitalizations demonstrated a significant decrease at the peak of the pandemic compared to 2019 and 2018 hospitalizations. The authors found a decreased number of admissions for septicemia, heart failure, myocardial infarction, and other acute presentations, many of which would have required imaging [22]. In our own hospital, nearly all patients were admitted for COVID-19; alternative diagnoses were much less common. We believe this to be the underlying etiology of the diminution of many of these studies. To our knowledge, there was no effort on the part of clinicians to reduce radiology studies in patients presenting for reasons other than COVID-19.

Our data demonstrate a decline in total thoracic CT utilization during the time of the pandemic, with the nadir of the 2020 trend line correlating with the first and second weeks of April. This concurs with the CDC recommendation against the use of chest CT for the diagnosis of COVID-19 infection [23]. Given the prevalence of thrombotic events in COVID-19 [24], we questioned whether CTA-PE studies might be increased during the pandemic. Our data did not support an increase in CTA-PE studies, showing a significant decrease in the daily number of studies. Research from a major New York City hospital and a major California hospital showed an overall decrease in emergency room utilization [25]. Given that CTA-PE studies obtained in the emergency department are a frequent metric for assessing trends in imaging, future data will likely include a “dip” in the trend lines of progressive increase in CTA-PE studies correlating with the pandemic. Of note, MR imaging studies for the first week of the pandemic were increased compared to the prior year; it is unclear if this represents a deliberate effort on the part of clinicians to perform studies before services were limited or if this was random variation. Importantly, we found that, after the acute phase of the pandemic, cardiothoracic imaging utilization returned to prepandemic levels, and cardiac imaging continued to increase despite the ongoing health crisis. There have been several important events during the chronic phase of the pandemic, including the approval of vaccines to prevent the disease, the introduction of several antiviral agents with anti-COVID-19 activity, and the emergence of successive COVID-19 variants, including the Delta and Omicron variants. Despite these events, only the initial emergence of COVID-19 had a significant impact on the utilization of cardiothoracic imaging. This has important implications: while many patients deferred care during the height of the pandemic, cardiothoracic radiology has recovered. Efforts to mitigate the effects of the pandemic can be focused on those patients who did not receive optimal care during the acute phase of the pandemic; however, patients currently presenting for cardiothoracic imaging are likely to receive the standard of care at this time.

The long-term effects of COVID-19 will impact cardiothoracic imaging for years to come. Studies have demonstrated progression to fibrotic lung disease in patients who have recovered from acute COVID-19 infection [26, 27], and some patients go on to require transplant [28]. Research has indicated that patients have not sought care for acute conditions such as acute coronary syndrome, and the long-term effects of deferred care may ultimately result in increased imaging studies for these patients [29, 30]. Furthermore, increasing evidence suggests the SARS-CoV-2 infection itself has long-term sequelae which may require long-term care requiring imaging [31]. In addition, radiologists experienced a substantial increase in workload as a result of the COVID-19 pandemic. A recent study conducted by Coppola et al. investigated the impact of the pandemic on members of the Italian Society of Medical and Interventional Radiology. The group found that most radiologists experienced a significantly reduced workload, leading to concerns that non-COVID-19 illnesses were not being appropriately diagnosed and treated, and the majority of radiology residents felt that their training was compromised due to the pandemic [32]. Several international studies have echoed these findings of altered training which may lead to suboptimal and poorly balanced education [33, 34], as have studies in other medical specialties [35, 36].

Limitations of this study included that it was performed at a single institution in a region of high community transmission. Improvements in patient management and available therapies will result in continued change in the response to COVID-19 infection and patient outcomes, which could not be accounted for in this retrospective study. Despite these limitations, these data provide a robust snapshot of cardiothoracic imaging during the pandemic and how it relates to trends in imaging over time.

## 5. Conclusions

The COVID-19 pandemic affected nearly all aspects of medicine, including imaging utilization in cardiothoracic radiology. Here, we quantify these changes and suggest clinical correlates as to why the observed changes occurred. While the COVID-19 pandemic significantly affected cardiothoracic imaging, our data demonstrate that it has rebounded and continues to grow as it did before the COVID-19 pandemic.

## Figures and Tables

**Table 1:** Summary of daily statistics.

	2019 daily average	SD	Median	2020 daily average	SD	Median	*p* value
Total radiographs per day	480	82.6	515	406	73.1	403	<0.0001
Portable radiographs	266	29.1	273.5	320	68.2	317.5	<0.0001
Total thoracic CT	52	21.4	61	21.8	12.9	19	<0.0001
Noncontrast CT	37.7	18.9	45.5	14.3	11.1	10.5	<0.0001
CTA-PE	14.1	4.4	14	7.5	4.4	7	<0.0001
Total cardiac CT	10.18	6.6	12.5	3.8	3.2	3	<0.0001
Calcium score CT	5.6	4.5	6	0.5	1	0	<0.0001
CTA-aortic dissection	1.6	1.2	1	0.08	0.3	0	<0.0001
Cardiac CTA	3.7	3.1	3	3.2	2.5	3	0.22
Total cardiac MRI	2.5	2.3	2	0.88	1.7	0	<0.0001

## Data Availability

The data presented in this manuscript are available from the corresponding author upon request.

## Supplementary Materials

Supplementary Materials STROBE checklist has been submitted as the supplemental material.
